# Efficient Whole-Cell Biocatalyst for Acetoin Production with NAD^+^ Regeneration System through Homologous Co-Expression of 2,3-Butanediol Dehydrogenase and NADH Oxidase in Engineered *Bacillus subtilis*

**DOI:** 10.1371/journal.pone.0102951

**Published:** 2014-07-18

**Authors:** Teng Bao, Xian Zhang, Zhiming Rao, Xiaojing Zhao, Rongzhen Zhang, Taowei Yang, Zhenghong Xu, Shangtian Yang

**Affiliations:** 1 The Key Laboratory of Industrial Biotechnology of Ministry of Education, School of Biotechnology, Jiangnan University, Wuxi, Jiangsu, People's Republic of China; 2 School of Biotechnology, Jiangnan University, Wuxi, Jiangsu, People's Republic of China; 3 School of Medicine and Pharmaceuticals, Jiangnan University, Wuxi, Jiangsu, People's Republic of China; 4 Department of Chemical Engineering, Ohio State University, Columbus, Ohio, United States of America; Virginia Tech, United States of America

## Abstract

Acetoin (3-hydroxy-2-butanone), an extensively-used food spice and bio-based platform chemical, is usually produced by chemical synthesis methods. With increasingly requirement of food security and environmental protection, bio-fermentation of acetoin by microorganisms has a great promising market. However, through metabolic engineering strategies, the mixed acid-butanediol fermentation metabolizes a certain portion of substrate to the by-products of organic acids such as lactic acid and acetic acid, which causes energy cost and increases the difficulty of product purification in downstream processes. In this work, due to the high efficiency of enzymatic reaction and excellent selectivity, a strategy for efficiently converting 2,3-butandiol to acetoin using whole-cell biocatalyst by engineered *Bacillus subtilis* is proposed. In this process, NAD^+^ plays a significant role on 2,3-butanediol and acetoin distribution, so the NADH oxidase and 2,3-butanediol dehydrogenase both from *B. subtilis* are co-expressed in *B. subtilis* 168 to construct an NAD^+^ regeneration system, which forces dramatic decrease of the intracellular NADH concentration (1.6 fold) and NADH/NAD^+^ ratio (2.2 fold). By optimization of the enzymatic reaction and applying repeated batch conversion, the whole-cell biocatalyst efficiently produced 91.8 g/L acetoin with a productivity of 2.30 g/(L·h), which was the highest record ever reported by biocatalysis. This work indicated that manipulation of the intracellular cofactor levels was more effective than the strategy of enhancing enzyme activity, and the bioprocess for NAD^+^ regeneration may also be a useful way for improving the productivity of NAD^+^-dependent chemistry-based products.

## Introduction

Acetoin (3-hydroxy-2-butanone, AC) is an extensively-used spice that naturally exists in corn, grape, cocoa, apple, butter, coffee, etc. Widely used in food and beverage industry, AC also serves as a platform compound in many other industries [1]. It is one of the 30 platform chemicals that are given priority to their development and utilization by the US Department of Energy [2]. Although there are many chemical synthetic methods for AC preparation [3], its market is limited by the disadvantages of traditional chemical synthesis. On the other hand, with the further development of green chemical technology and the constant improvement of the environmental protection consciousness, non-toxic and non-pollution biological technology inevitably become the main direction of industrial development and consumers prefer security natural products even though they are generally more expensive than the corresponding chemical compounds.

Nowadays, a lot of efforts have been made to develop natural AC production using fermentative [4], enzymatic [5] or biocatalytic technologies [6]. A number of bacteria have abilities to produce AC, including the genera *Klebsiella*, *Paenibacillus*, *Gluconobacter*, *Bacillus*, *Serratia*, etc. [7]–[11]. However, in most of these species, AC just plays a part as by-product of 2,3-butanediol (2,3-BD), which is another important bio-based platform chemical [12]. *Bacillus* species, which can produce various of industrial products [13], have been proved with AC as its major fermentation product under specific conditions [14]. Many efforts have been made to improve the production of AC from *Bacillus* strains. Liu et al. isolated a *B. licheniformis* strain that could produce 41.3 g/L of AC [4]. Zhang et al. isolated the *B. subtilis* JNA-3-10 and produced 42.2 g/L of AC [15]. Fermentation optimization strategies have been used to improve AC production, such as optimizing the medium components [16], controlling the level of dissolved oxygen and controlling the fermentation pH [17]. Metabolic engineering strategies were also applied to improve AC production through modifying metabolic branchpoints in the network [14], [18], [19]. However, so much long fermentation duration lead to a low AC productivity. To our knowledge, the highest productivity of AC by *Bacillus* strains is just 1.42 g/(L·h) [4]. In addition, the mixed acid-butanediol fermentation of *Bacillus* strains will metabolize a certain portion of sugars to the by-products of organic acids such as lactic acid and acetic acid, which causes energy cost and increases the difficulty of product purification in downstream processes [20].

Recently, the introduction of NAD^+^ regeneration system could dramatically improve AC production and decrease the yield of NADH-dependent by-products [6], [11]. Sun et al. obtained 75.2 g/L AC with a productivity of 1.88 g/(L·h) by *S. marcescens* H32 with over-expression of a water-forming NADH oxidase [11]. Xiao et al. developed a co-expression system with 2,3-butanediol dehydrogenase and NADH oxidase in *E. coli* produced AC at a high productivity of 3.06 g/(L·h) [6]. Although have relative high AC productivities, AC yield of this biocatalyst was still far away from the highest report of 89.2 g/L achieved by Wang et al. using fermentation method with 2,3-BD as substrate by *Gluconobacter oxydans* DSM 2003 [10]. Therefore, combining both advantages of fermentation and cofactor regeneration, a potential strategy of introducing a biocatalytic process with NAD^+^ regeneration system for efficient natural AC production in *B. subtilis* is proposed by us. Whole-cell biocatalyst has been intensively explored for the production of valuable compounds because excellent selectivity and NAD^+^ reserves that provides a continuous source of cofactors [21]. Trough NAD^+^ regeneration system, the cellular cofactor level, redox state and the corresponding enzymatic activity are expected to have major effects on the performance of the whole-cell biocatalysts. In this whole-cell biocatalyst, 2,3-BD is used as substrate and only AC can be obtained in the short biocatalyst period. This is also a good solution to develop derivative process for industrially produced 2,3-BD utilization, which can not be commercially utilized so far.

**Figure 1:**
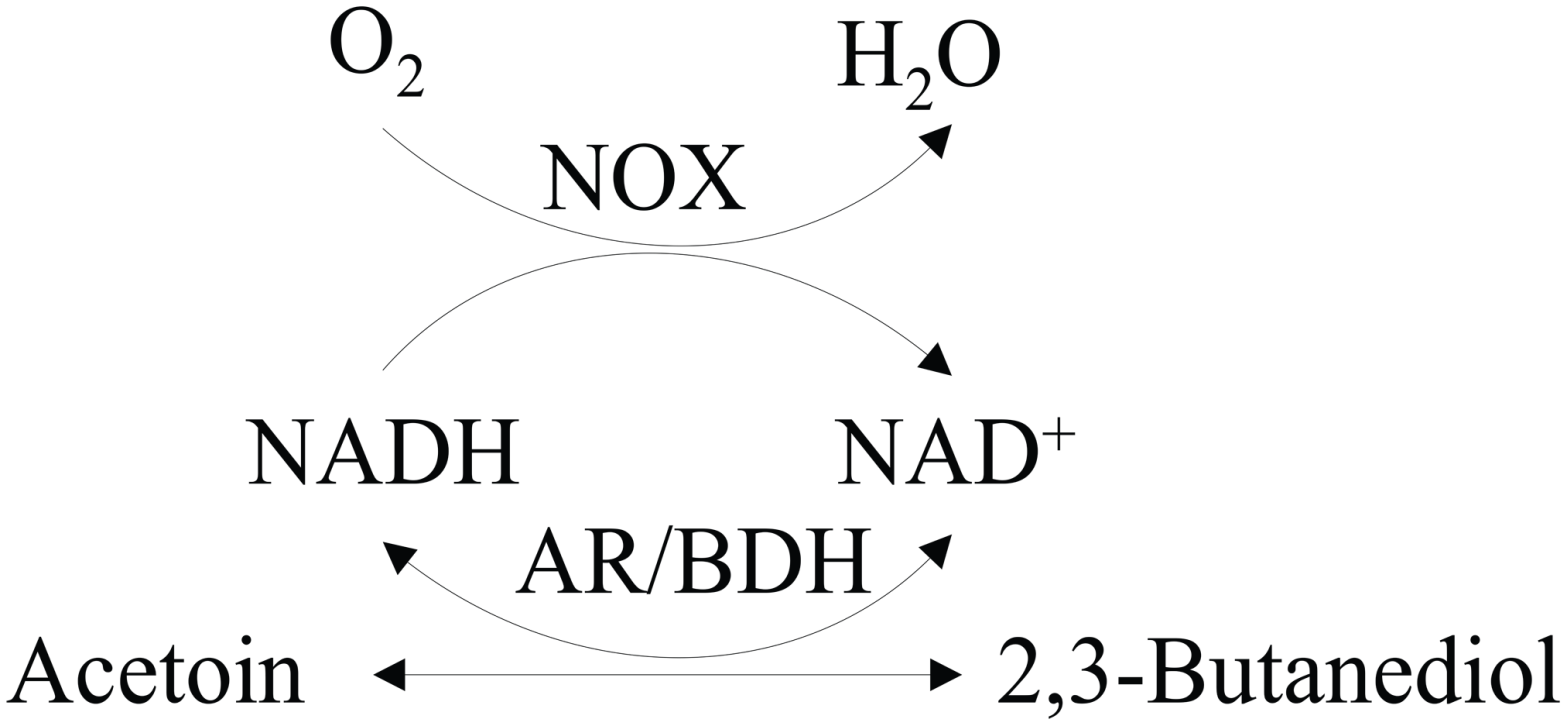
Whole-cell biocatalyst with NAD^+^ regeneration system for the production of acetoin. AR/BDH: acetoin reductase/2,3-butanediol dehydrogenase; NOX: NADH oxidase.

In previous work of our lab, when *B. subtilis* was fermented with glucose as substrate, 2,3-BD was the major product in prophase of fermentation, whereas in the anaphase of fermentation, 2,3-BD was reversely transformed to AC [15]. The reversible conversion between AC and 2,3-BD was responsible by the enzyme AC reductase/2,3-BD dehydrogenase (AR/BDH EC 1.1.1.4). Generally, AR/BDHs have the similar optimum pH-values for oxidation and reduction [22]–[24]. However, very special property of this enzyme that has different optimum pH-values for oxidation and reduction was proved [25], [26]. Intrigued by this point, our group experimentally demonstrated that AR/BDH from *B. subtilis* also has very different optimum pH-values [27]. The results indicated that this enzyme preferentially catalyzes the reduction/oxidation reaction in the acidic/alkaline condition. Similar to other reductases and dehydrogenases, AR/BDH requires NAD^+^ and NADH as cofactors [28], [29]. As one pair of key cofactors, NADH and NAD^+^ play a critical role in over 300 biochemical reactions including oxidation and reduction [30], [31], which helps to maintain the elementary requirement for metabolism and growth in cells [32]. During the catalytic process of BDH, NAD^+^ is reduced to NADH accompanied by the oxidization of 2,3-BD [6]. Therefore, an driving force performed by NADH oxidase (NOX EC 1.6.99.3) was required for sweeping away the obstruction proposed on NAD^+^ regeneration. As a flavoprotein, NOX uses oxygen to produce either water or hydrogen peroxide [33], which can partially or completely inhibit cell growth. In this work, the homologous NOX from *B. subtilis*, similar to the NOX from Lactobacillus brevis [33], generates NAD^+^ from NADH by reducing O_2_ to H_2_O was used as the core enzyme for NAD^+^ regeneration [34], because construction of a homologous NAD^+^ regeneration system can be more efficient and safe for AC production. The constructed biocatalyst of *B. subtilis*, in which AR/BDH and NOX were co-expressed (shown in Figure 1), adjusted the intracellular NADH/NAD^+^ ratio and NAD(H) level and strongly pumped the catalytic reaction. After optimization of the biocatalytic conditions, AC production and productivity was highly improved.

## Materials and Methods

### Chemicals, media and cultivation

Ampicillin, kanamycin, NAD^+^, NADH, FAD, dithiothreitol (DTT) and phenylmethanesulfonyl fluoride (PMSF) were obtained from Sangon Biotech (Shanghai, China). PCR primers were synthesized by Sangon Biotech (Shanghai, China). The restriction endonucleases and T4 DNA ligase were obtained from Takara (Dalian, China). All other chemicals were commercially available reagents of analytical grade.

*B. subtilis* and *E. coli* were cultivated in Luria-Broth (LB) medium routinely and were used as the cloning and expression hosts, respectively. When necessary, antibiotics (100 µg/mL ampicillin or 50 µg/mL kanamycin) were supplemented to the medium to maintain the plasmids.

### Strains, plasmids and primers

The strains, plasmids and primers used in this study were given in Table 1.

**Table 1:** Bacterial strains, plasmids and primers used.

Strains/plasmids/primers	Characteristics	Sources
**Strains**		
*Escherichia coli*		
* E. coli* JM109	*recA1, endA1, gyrA96, thi-1, hsd R17(r_k_^−^ m_k_^+^)supE44*	Invitrogen
* E. coli* JM109/ pMA5-*bdhA*	*E. coli* JM109 containing pMA5-*bdhA* (Amp^R^)	This study
* E. coli* JM109/ pMA5-*yodC*	*E. coli* JM109 containing pMA5-*yodC* (Amp^R^)	This study
* E. coli* JM109/ pMA5-*bdhA*-*yodC*	*E. coli* JM109 containing pMA5-*bdhA*-*yodC* (Amp^R^)	This study
*Bacillus subtilis*		
* B. subtilis* 168	*trpC2*	Laboratory stock
* B. subtilis* 168/ pMA5	*B. subtilis* 168 containing pMA5 (Km^R^)	Laboratory stock
* B. subtilis* 168/ pMA5-*bdhA*	*B. subtilis* 168 containing pMA5- *bdhA* (Km^R^)	This study
* B. subtilis* 168/ pMA5-*yodC*	*B. subtilis* 168 containing pMA5-*yodC* (Km^R^)	This study
* B. subtilis* 168/ pMA5-*bdhA*-*yodC*	*B. subtilis* 168 containing pMA5-*bdhA*-*yodC* (Km^R^)	This study
**Plasmids**		
pMA5	*Hpa*II promoter, colE1 ori, *repB*, replicates in *E. coli* (Amp^R^) or *B. subtilis* (Km^R^)	Laboratory stock
pMA5-*bdhA*	pMA5 containing *bdhA*-His	This study
pMA5-*yodC*	pMA5 containing *yodC*-His	This study
pMA5-*bdhA-yodC*	pMA5 containing *bdhA*-His and *yodC*-His	This study
**Primers 5**′**-3**′		
P1	ACCGGGATCCATGAAGGCAGCAAGATGG (*Bam*H I)	
P2	ACCGACGCGTTTAGTGGTGGTGGTGGTGGTGGTTAGGTCTAACAAGG (*Mlu* I)	
P3	ACCGGGATCCATGACGAATA CTCTGGAT (*Bam*H I)	
P4	ACCGACGCGTTTAGTGGTGGTGGTGGTGGTGCAGCCAA GTTGATAC (*Mlu* I)	
P5	ACCGGCATGCGTAAGCTAGACAAAACGGAC (*Sph* I)	
P6	ACCGAAGCTTTTAGTGGTGGTGGTGGTGGTGCAGCCAAGTTGATAC (*Hind* III)	

Amp^R^ ampicillin-resistant, Km^R^ kanamycin-resistant, the restriction enzyme sites were bold typed, and the His-Taq coding region were underlined.

### Construction of recombinant plasmids

The *bdhA* and *yodC* genes were amplified using forward primer P1/P3 and reverse primer P2/P4 and *B. subtilis* 168 as template. The plasmid pMA5-*bdhA*/pMA5-*yodC* were constructed by inserting *bdhA/yodC* between the *Bam*H I and *Mlu* I sites of pMA5. The *yodC* gene with with the upstream region including *Hpa*II promoter and RBS was then amplified using forward primer P5 and reverse primer P6 and pMA5-*yodC* as template. The plasmid pMA5-*bdhA-yodC* was constructed by inserting *Hpa*II-*yodC* between the *Sph* I and *Hind* III sites of pMA5-*bdhA*.

### Transformation and selection of recombinant strains

The ligated DNAs were used to transform *E. coli* JM109. Positive colonies were selected on agar plates containing ampicillin, and the plasmids were confirmed using restriction enzyme analysis and DNA sequencing. The recombinant plasmids were then used to transform *B. subtilis* 168 [35]. The transformants were screened for their ability to grow on LB agar plates containing kanamycin.

### Assays of AR/BDH and NOX activities and SDS-PAGE analysis

The cells were harvested by centrifugation at 10000 rpm for 10 min at 4°C and washed twice with 50 mM sodium phosphate buffer (pH 6.5). Then the harvested cells were resuspended in 50 mM sodium phosphate buffer (pH 6.5) containing 0.1 mM PMSF, and 1 mM DTT. After ultrasonic disruption, cell debris was removed by centrifugation at 15000 rpm at 4°C for 30 min to obtain a crude enzyme solution for the enzyme assay.

The AR/BDH and NOX activities were determined spectrophotometrically by measuring the change of absorbance at 340 nm and 25°C corresponding to the oxidation of NADH (ε_340_ = 6220/M·cm) or the reduction of NAD^+^. AR activity was determined using 50 mM sodium phosphate buffer (pH 6.5) containing 50 mM acetoin and 5 mM NAD^+^. BDH activity was determined using 50 mM glycine-NaOH buffer (pH 8.5) containing 100 mM 2,3-butanediol and 5 mM NADH. One unit of activity (U) corresponds to 1 µmol NAD(H) formed per minute [23].

NOX activity was determined using 50 mM sodium phosphate (pH 7.0) containing 0.3 mM EDTA, 50 µM FAD and 0.3 mM NADH. One unit of activity (U) corresponds to 1 µmol NAD^+^ formed per minute [36].

The enzymes were assayed by sodium dodecyl sulfate-polyacrylamide gel electrophoresis (SDS-PAGE) according to Laemmli method [37]. AR/BDH and NOX were also detected by Western blotting by using a mouse monoclonal anti-His_6_ antibody. The extracts were loaded in a 12% SDS-PAGE gel that was blotted onto a polyvinylidene difluoride (PVDF) membrane (Millipore) and treated with the primary anti-His_6_ antibody. To visualize the relevant bands, the blot was treated with a secondary goat anti-mouse antibody (Bio-Rad) conjugated to horseradish peroxidase (HRP), and the bands were detected by chemiluminescence with luminol and peroxide with the aid of a Bio-Rad Chemidoc XRS. The molecular weight of the subunit of AR/BDH and NOX were determined by comparing the relative mobility of perfect protein Marker 14.3–97.2 kDa (Takara). The protein concentration was determined by Bradford method [38] using BSA as the standard protein.

### Biocatalyst preparation and biocatalysis conditions

*B. subtilis* cells were grown at 37°C with fermentation medium (soya peptone 1 g/L, glucose 4 g/L, corn steep liquor 1.5 g/L, urea 0.3 g/L, K_2_HPO_4_·3H_2_O 0.17 g/L, KH_2_PO_4_ 0.23 g/L, MnSO_4_·H_2_O 0.075 g/L, NaCl 5 g/L, pH 6.8–7.0) in shake flasks. After cultivation for 36 h (OD_600_ = 13.0–16.0), the cells were harvested by centrifugation at 10000 rpm for 10 min at 4°C and washed twice with 50 mM sodium phosphate buffer (pH 6.5). Then 500 mL cell cultures were resuspended into 50 mL 50 mM sodium phosphate buffer (pH 8.0) containing 40 g/L 2,3-BD. The whole-cell biocatalysis was performed on a rotary shaker (200 rpm) at 37°C.

When whole-cell biocatalysis carried out in a 5-L fermentor (Baoxing Co., Shanghai, China), 20 L of recombinant *B. subtilis* cultures was harvested and resuspended into 2 L of 50 mM sodium phosphate buffer (pH 8.5) containing 40 g/L 2,3-BD and 5 mM MnCl_2_. The agitation speed of the whole-cell biocatalysis was 200 rpm.

The whole-cell biocatalytic activity was assayed by measuring the formation of AC, and one unit was defined as 1.0 mM AC produced per hour per OD_600_ of culture at 37°C.

### Determination of NADH and NAD^+^ concentrations

The intracellular concentrations of NADH and NAD^+^ were determined using the Amplite Fluorimetric NAD/NADH Ratio Assay Kit (15263) from AAT Bioquest (Sunnyvale, CA, USA) according to the manufacturers' instructions. The assay kit provides a convenient method for sensitive detection of NAD^+^, NADH and their ratio. The signal can be easily read by either a fluorescence microplate reader at Ex/Em = 530–570/590–600 nm (maximum Ex/Em = 540/590 nm) or an absorbance microplate reader at ∼576 nm [39].

### Optimization of the whole-cell biocatalyst

The following buffer systems were used to investigate the optimal pH of the biocatalyst: 50 mM acetate-sodium acetate buffer (pH 4.5–5.5), 50 mM sodium phosphate buffer (pH 5.5–8.5) and 50 mM glycine-NaOH buffer (pH 8.5–10.5). Conversion temperature (20–50°C), concentration of substrate (10–60 g/L) and the concentration of metal ion stimulaters (MgCl_2_, MnCl_2_, CaCl_2_, FeCl_2_ and FeCl_3_) were also optimized.

### Analysis methods

The bio-profiles were monitored periodically over the experiment period. Samples were centrifuged at 10000 rpm for 10 min, and the supernatant was used for further analysis. Concentrations of AC and 2,3-BD were monitored by gas chromatography (Jie Dao GC1600, China, FID detector, N_2_ flow rate of 50 ml/min, detector temperature of 250°C, and a column temperature of 160°C). Biomass was measured at OD_600_ (UNICO UV-2000) of the culture broth after appropriate dilution with water. All assays were performed by triplicate cultures. The samples were determined at least twice for three extracts derived from independent cultures, and standard deviations of the biological replicates were represented by error bars.

## Results and Discussion

### Construction of the whole-cell biocatalyst

**Figure 2:**
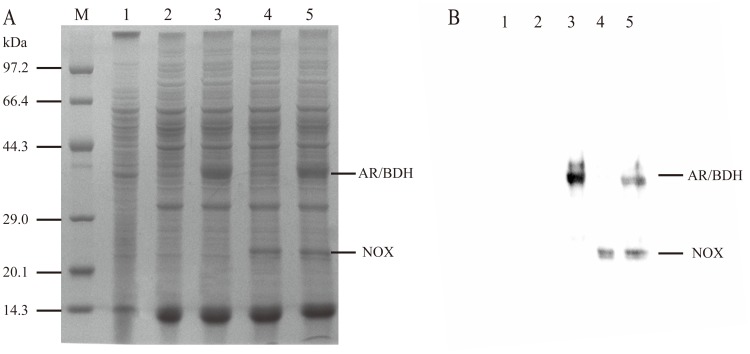
Expression analysis of recombinant *B. subtilis*. (A) SDS-PAGE result of AR/BDH and NOX expressed in *B. subtilis*. Lane M, protein marker; lane 1, *B. subtilis* 168; lane 2, *B. subtilis* 168/pMA5; lane 3, *B. subtilis* 168/pMA5-*bdhA*; lane 4, *B. subtilis* 168/pMA5-*yodC*; lane 5, *B. subtilis* 168/pMA5-*bdhA*-*yodC.* (B) western blot result of AR/BDH and NOX expressed in *B. subtilis*. lane 1, *B. subtilis* 168; lane 2, *B. subtilis* 168/pMA5; lane 3, *B. subtilis* 168/pMA5-*bdhA*; lane 4, *B. subtilis* 168/pMA5-*yodC*; lane 5, *B. subtilis* 168/pMA5-*bdhA*-*yodC.*

To construct the whole-cell biocatalyst, the following strains of *B. subtilis* 168, *B. subtilis* 168/pMA5, *B. subtilis* 168/pMA5-*bdhA*, *B. subtilis* 168/pMA5-*yodC* and *B. subtilis* 168/pMA5-*bdhA-yodC* were detected to their enzyme activities and whole-cell biocatalytic activities. The results were shown in Table 2. *B. subtilis* 168/pMA5-*bdhA* and *B. subtilis* 168/pMA5-*yodC* showed the BDH and NOX activity of 124.5 mU/mg and 570.3 mU/mg, respectively. As expected, the co-expression system of *B. subtilis* 168/pMA5-*bdhA*-*yodC* showed a BDH activity of 152.8 mU/mg and a NOX activity of 346.4 mU/mg. SDS-PAGE analyze of the recombinant protein expression was shown in Figure 2. AR/BDH and NOX have the specific molecular weights of 37.3 kDa and 25.7 kDa, respectively. The results of SDS-PAGE and enzyme activity assay indicated that *bdhA* were successfully co-expressed with *yodC*.

**Table 2:** Enzyme activity and the whole-cell biocatalytic activity of different strains.

Strains	AR activity (mU/mg)	BDH activity (mU/mg)	NOX activity (mU/mg)	Whole-cell biocatalytic ability (U/L)
*B. subtilis* 168	40.2±0.96	1.9±0.03	33.4±1.03	14.10±0.41
*B. subtilis* 168/pMA5	38.3±0.84	1.5±0.04	31.6±0.73	15.21±0.44
*B. subtilis* 168/pMA5-*bdhA*	179.7±5.21	124.5±3.11	34.9±0.94	18.24±0.42
*B. subtilis* 168/pMA5-*yodC*	63.5±1.71	4.8±0.11	570.3±18.25	16.55±0.51
*B. subtilis* 168/pMA5-*bdhA*-*yodC*	171.8±5.32	152.8±4.12	346.4±9.70	24.12±0.53

In comparison with free enzyme activities, the whole-cell biocatalytic activities prepared from the recombinants were defined as the formation of 1.0 mmol AC per hour per OD_600_ of culture at 37°C, which were much more direct to prove the biocatalytic abilities. As shown in Table 1, *B. subtilis* 168 could not effectively catalyze 2,3-BD to AC because of low AR/BDH activity and the restriction of NAD^+^ pool. By over-expression of AR/BDH or NOX, the whole-cell biocatalytic activities of *B. subtilis* 168/pMA5-*bdhA* and *B. subtilis* 168/pMA5-*yodC* were both increased, but no more than 30 % compared to *B. subtilis* 168. However, the whole-cell biocatalytic activity of *B. subtilis* 168/pMA5-*bdhA*-*yodC* was nearly twice that of *B. subtilis* 168, indicating the NAD^+^ regeneration system worked successfully.

Although AR/BDH activity of *B. subtilis* 168/pMA5-*yodC* was lower than *B. subtilis* 168/pMA5-*bdhA*, the over-expressed NOX regenerated sufficient NAD^+^ to catalyze 2,3-BD to AC. So both *B. subtilis* 168/pMA5-*yodC* and *B. subtilis* 168/pMA5-*bdhA*-*yodC* showed obviously increased whole-cell biocatalytic activities by over-expressing of an NAD^+^ regeneration enzyme, suggesting cofactor regeneration is more significant than improving the catalytic enzyme activity.

### Effects of intracellular NADH and NAD^+^ concentrations on AC biosynthesis

**Figure 3:**
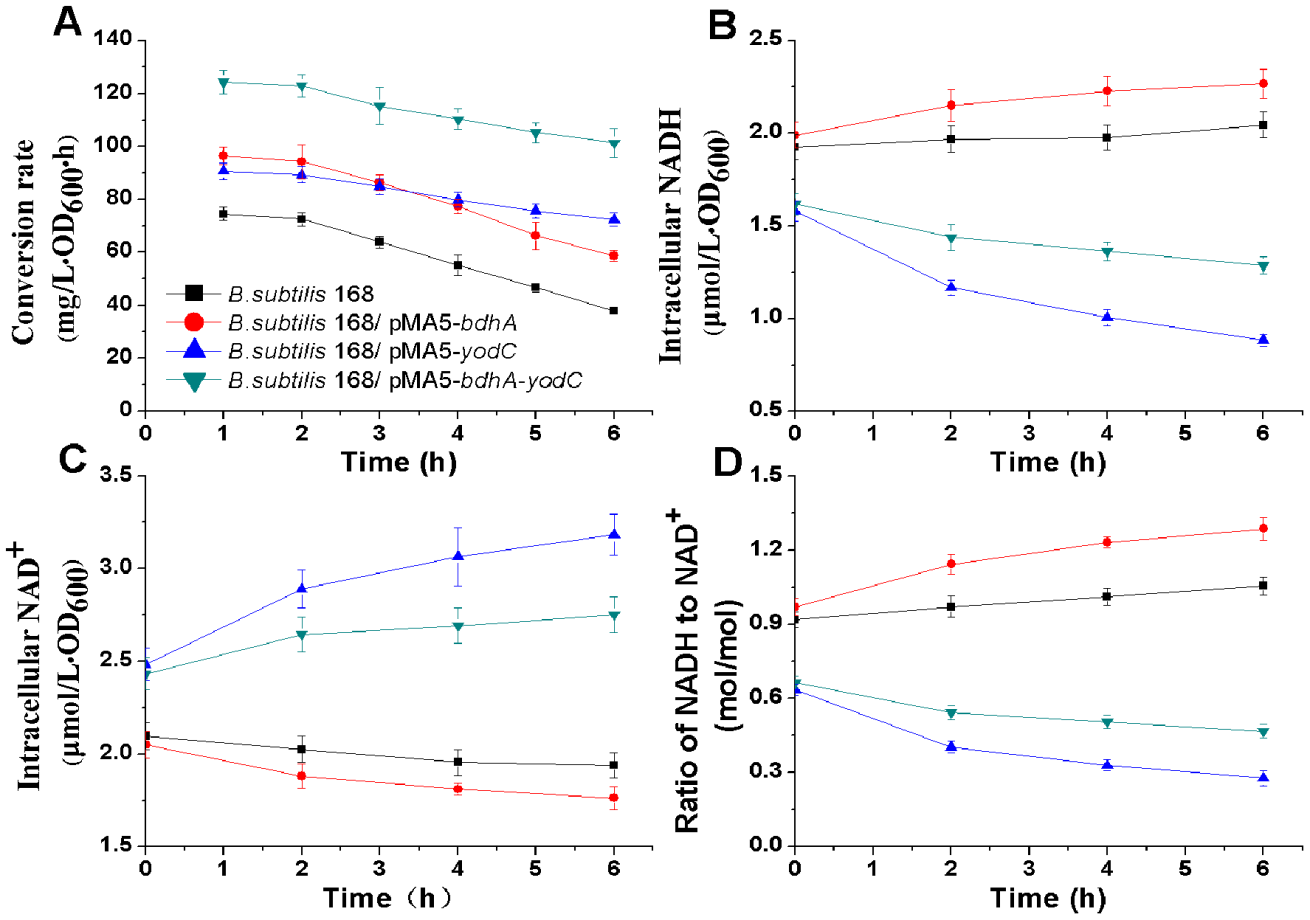
The intracellular NADH, NAD^+^ concentrations in different biocatalysts and the effect of NADH/NAD^+^ ratio on conversion rate.

The recombinant strains and the parent strain were cultured in the fermentation medium. Homologous over-expression of NOX in *B. subtilis* was expected to regenerate the overall intracellular NAD^+^ pool for continuously converting AC to 2,3-BD. As shown in Figure 3A, *B* *. subtilis* 168/pMA5-*bdhA*-*yodC* achieved the highest conversion rate of 2,3-BD to AC comparing to the other biocatalysts. Both *B. subtilis* 168/pMA5-*yodC* and *B. subtilis* 168/pMA5-*bdhA*-*yodC* could maintain efficient productivities to catalyze 2,3-BD to AC, indicating NAD^+^ was regenerated by NOX.

The significant role of cofactors played in these biocatalysts was further proved by comparing the intracellular concentrations of NAD^+^ and NADH in the recombinants, in which the overall amount of intracellular NAD^+^ and NADH were almost constant within 6 hours. The intracellular NAD^+^ pools in recombinant *B. subtilis* 168/pMA5-*bdhA*-*yodC* and *B. subtilis* 168/pMA5-*yodC* were greatly improved by over-expression of NOX (Figure 3B and 3C), and NAD^+^ was continuously regenerated to keep a persistent AC productivity. Although over-expression of AR/BDH by *B. subtilis* 168/pMA5-*bdhA* effectively converted 2,3-BD to AC in the first 2 hours, the reduced NAD^+^ pool then restricted its process for the bioconversion.

By comparing of the intracellular NAD^+^ and NADH levels and the ratio of NADH to NAD^+^ (Figure 3D), NAD^+^ was proved be regenerated by over-expression of NOX. After 6 h, the intracellular NAD^+^ concentration of *B. subtilis* 168/pMA5-*bdhA*-*yodC* increased to 2.75 µmol/(L·OD_600_), higher than *B. subtilis* 168 and *B. subtilis* 168/pMA5-*bdhA*. Corresponding to above results, the intracellular NADH concentration of *B. subtilis* 168/pMA5-*bdhA*-*yodC* decreased to 1.29 µmol/(L·OD_600_), lower than *B. subtilis* 168 and *B. subtilis* 168/pMA5-*bdhA*. Although *B. subtilis* 168/pMA5-*yodC* achieved the highest NOX activity, which resulted in the lowest NADH/NAD^+^ ratio, it did not showed the highest conversion rate from 2,3-BD to AC. The results suggested that a relatively stable dynamic redox balance of low NADH/NAD^+^ ratio as well as a high AR/BDH activity were necessary for efficiently and persistently converting 2,3-BD to AC. So that, the biocatalyst of *B. subtilis* 168/pMA5-*bdhA*-*yodC*, which co-expressed AR/BDH and NOX was chosen for later experiments.

### Optimization of the biocatalytic reaction

**Figure 4:**
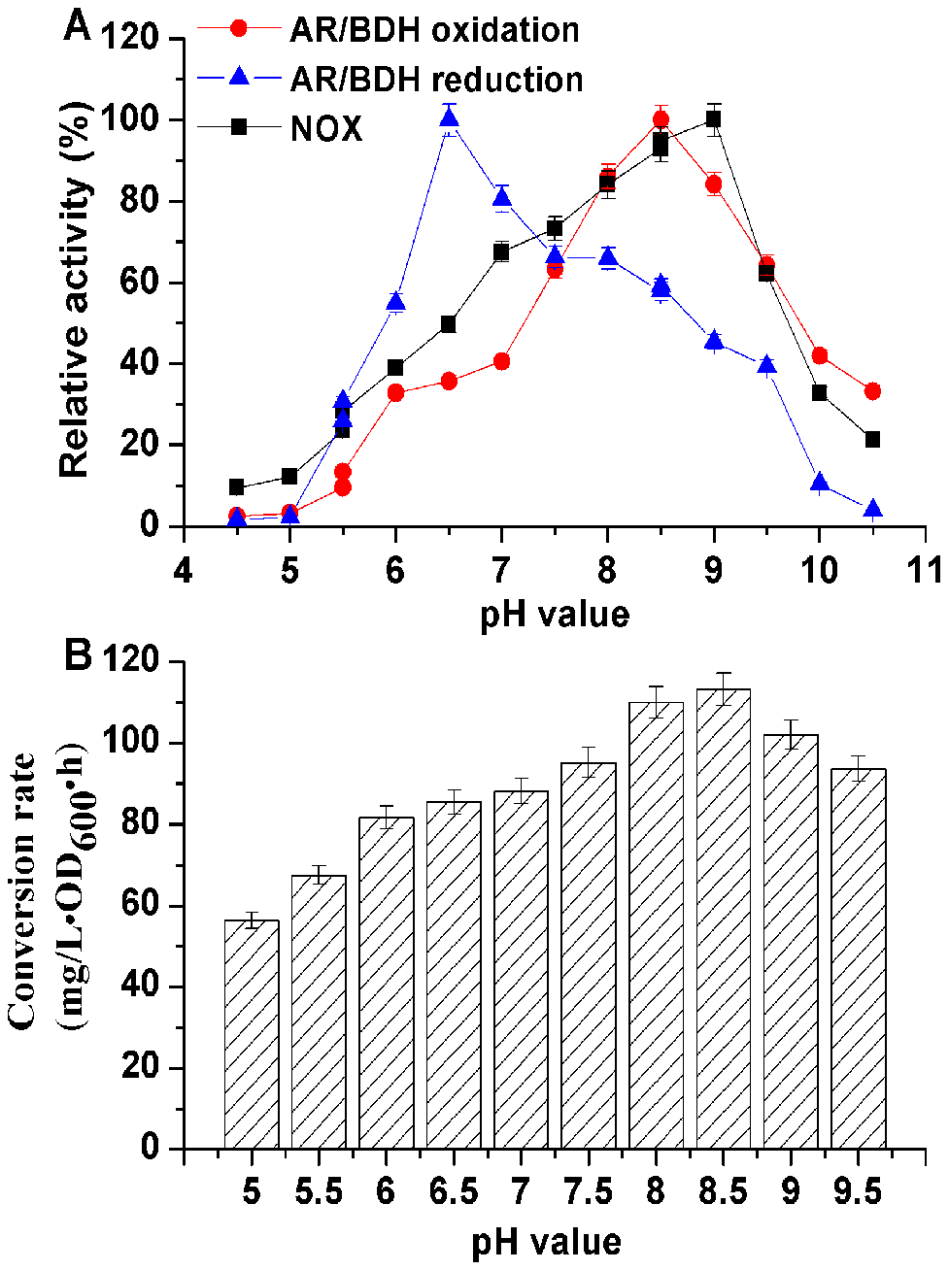
Effect of pH on the conversion rate of whole-cell biocatalyst. (A) Enzyme activities in different pH-values; (B) whole-cell biocatalyst conversion rate.

To achieve higher productivity, the whole-cell biocatalytic reaction was optimized. As an important parameter that often limit enzyme activity and stability in technical applications [40], the effect of pH on the conversion rate of the biocatalyst was investigated. As shown in Figure 4A, AR/BDH had different optimum pH-values of 8.5 and 6.5 for oxidation and reduction, respectively. Meanwhile, the optimum pH value for NOX was 9.0, and this enzyme could maintain 80 % relative activity under pH 8.0–9.0. So the efficiency of the whole-cell biocatalyst could be affected by adjusting the pH-values of the conversion solution. As shown in Figure 4B, reactions with 40.0 g/L of 2,3-BD as substrate in the biocatalyst *B. subtilis* 168/pMA5-*bdhA*-*yodC* were conducted under different pH-values for 6 h. The conversion rate was measured by monitoring the yield of AC. The results indicated that the conversion rate of this biocatalyst was gradually increased by improving the pH levers in the alkaline condition and the highest conversion rate was acquired at pH 8.5. Thus, we use pH 8.5 as the optimum conversion pH-value for the following experiments.

**Figure 5:**
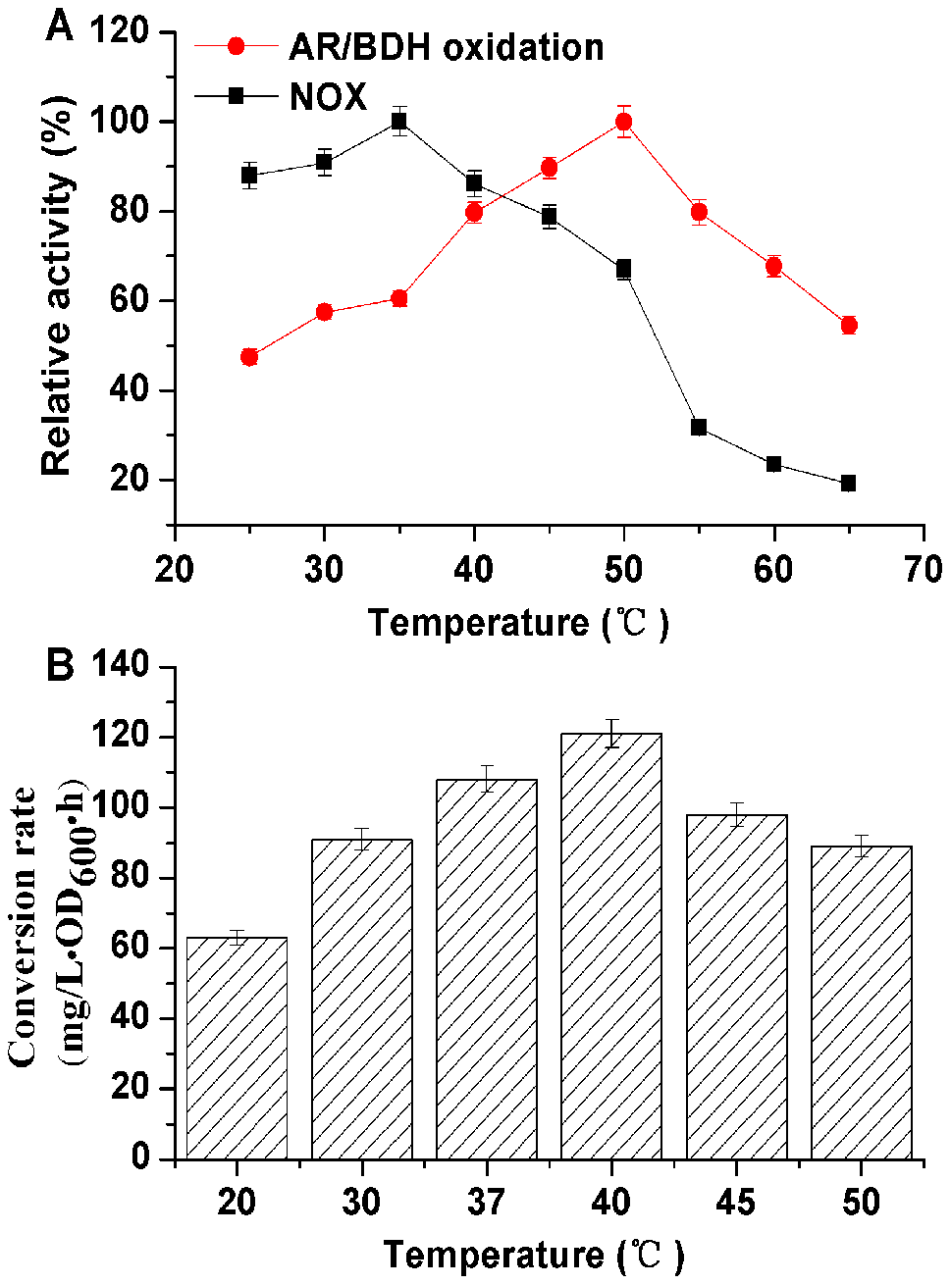
Effect of temperature on the conversion rate of whole-cell biocatalyst. (A) Enzyme activities under different temperature; (B) whole-cell biocatalyst conversion rate.

Temperature can also affect the efficiency of the whole-cell catalytic processes, including enzyme activity and cellular maintenance [10]. Thus, we studied the effect of temperature on AC conversion rate using this biocatalyst. As shown in Figure 5A, AR/BDH and NOX had different optimum temperature. The optimum oxidation temperature for AR/BDH activity was 50°C (similar to the optimum reduction temperature, data was not shown), but the optimum temperature for NOX activity was 35°C. However, both enzymes could maintain 80 % relative activities under the temperature 40–45°C. So the whole-cell bioconversion was assayed under the following temperatures of 20°C, 30°C, 37°C (the best growing temperature), 40°C, 45°C and 50°C, respectively (Figure 5B). The results indicated that the highest conversion rate of this biocatalyst was obtained at a relatively high temperature 40°C, closed to the best growing temperature. Finally, a temperature of 40°C was chosen for the optimum temperature.

**Figure 6:**
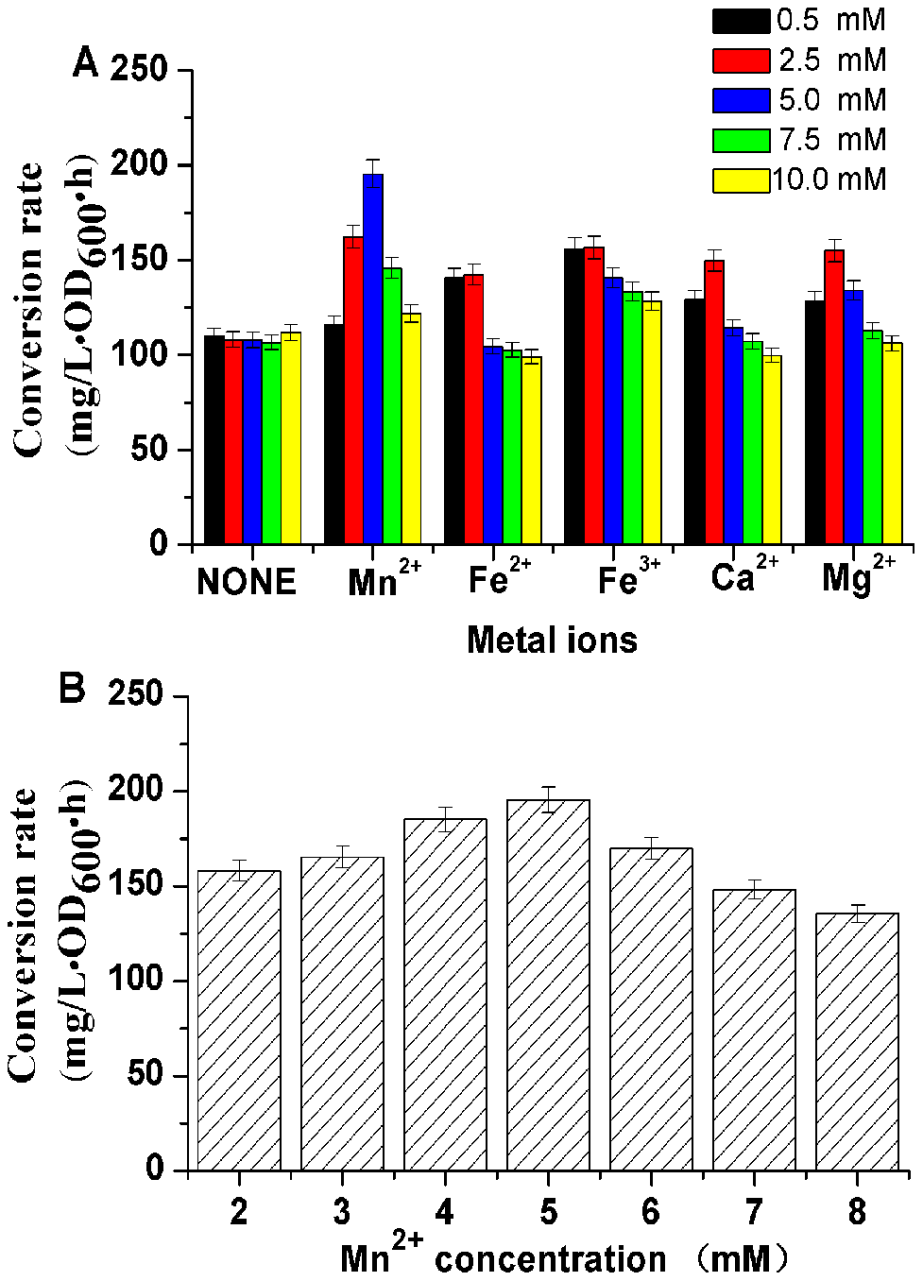
Effect of metal ion stimulators on the conversion rate of whole-cell biocatalyst. (A) Effect of different metal ions on the conversion rate of the biocatalyst; (B) Effect of Mn^2+^ concentration on the conversion rate of the biocatalyst.

Chemical stimulators such as Mg^2+^, Mn^2+^ and Ca^2+^ can improve the activity of AR/BDH [27]. However, another enzyme in this biocatalyst, NOX, had been proved that mental ions have neither stimulation effect nor inhibition effect on its activity [34]. Thus, the following chemicals of MgCl_2_, MnCl_2_, CaCl_2_, FeCl_2_ and FeCl_3_ were studied of their effect on the biocatalyst, and they were added to the conversion solution at the final concentrations of 0.5 mM, 2.5 mM, 5.0 mM, 7.5 mM and 10.0 mM, respectively. Of all the chemicals listed in Figure 6A, 2.5–5.0 mM Mn^2+^ showed remarkable stimulation effect on AC conversion rate. Other metal ions just slightly stimulated the convention rate of 2,3-BD to AC. To find the optimum Mn^2+^ concentration on the efficiency of this biocatalyst, the final concentration of Mn^2+^ was adjusted from 2.0 mM to 8.0 mM (Figure 6B). The results indicated that 5.0 mM Mn^2+^ was favorable for this whole-cell biocatalyst.

**Figure 7:**
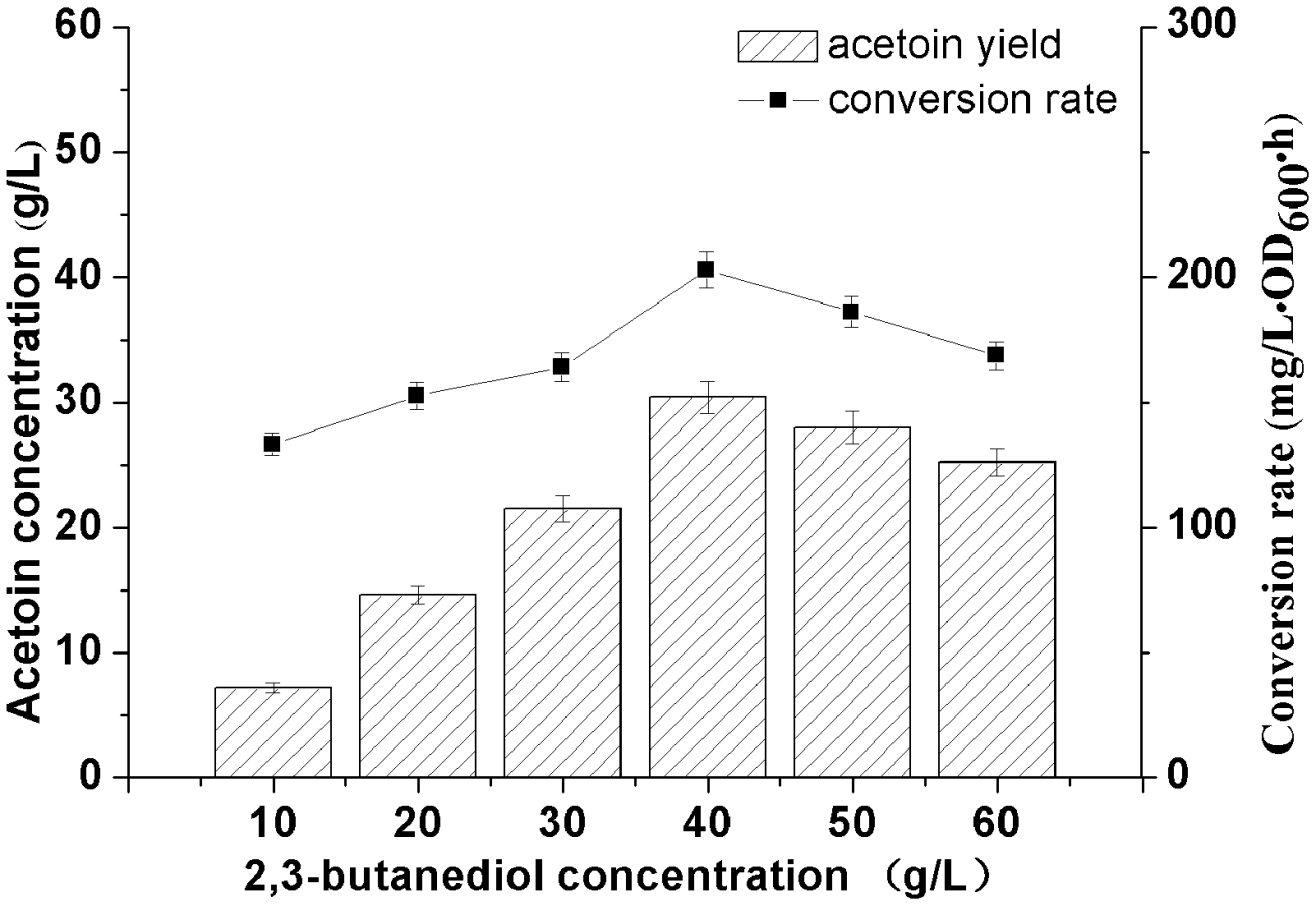
Effect of 2,3-butanediol concentration on the conversion rate of whole-cell biocatalyst.

Substrate concentration is another important factor in whole-cell catalytic processes, which may result in substrate inhibition. The effect of 2,3-BD concentration on the conversion rate using the biocatalyst was tested (Figure 7). The results showed that the biocatalyst achieved the highest conversion rate with 40 g/L 2,3-BD as substrate. While 2,3-BD concentration kept on increasing, the conversion rate could be inhibited by high concentration of substrate [6]. AR/BDH can catalyze the stereospecific oxidation of (2R,3R)-2,3-BD and meso-2,3-BD to (3R)-AC and (3S)-AC, respectively [41]. However, it cannot catalyze the conversion of (2S,3S)-2,3-BD. Thus, while using 40.0 g/L of mixed stereospecific 2,3-BD as substrate, the highest AC yield was 31.5 g/L after 12 h. Although 2,3-BD could not be totally converted into AC, it can be easily separated from AC which has a low boiling point. Therefore, 40.0 g/L of 2,3-BD was chosen as the optimum substrate concentration for this biocatalyst.

### Whole-cell biocatalyst using repeated batch strategy under optimized conditions

**Figure 8:**
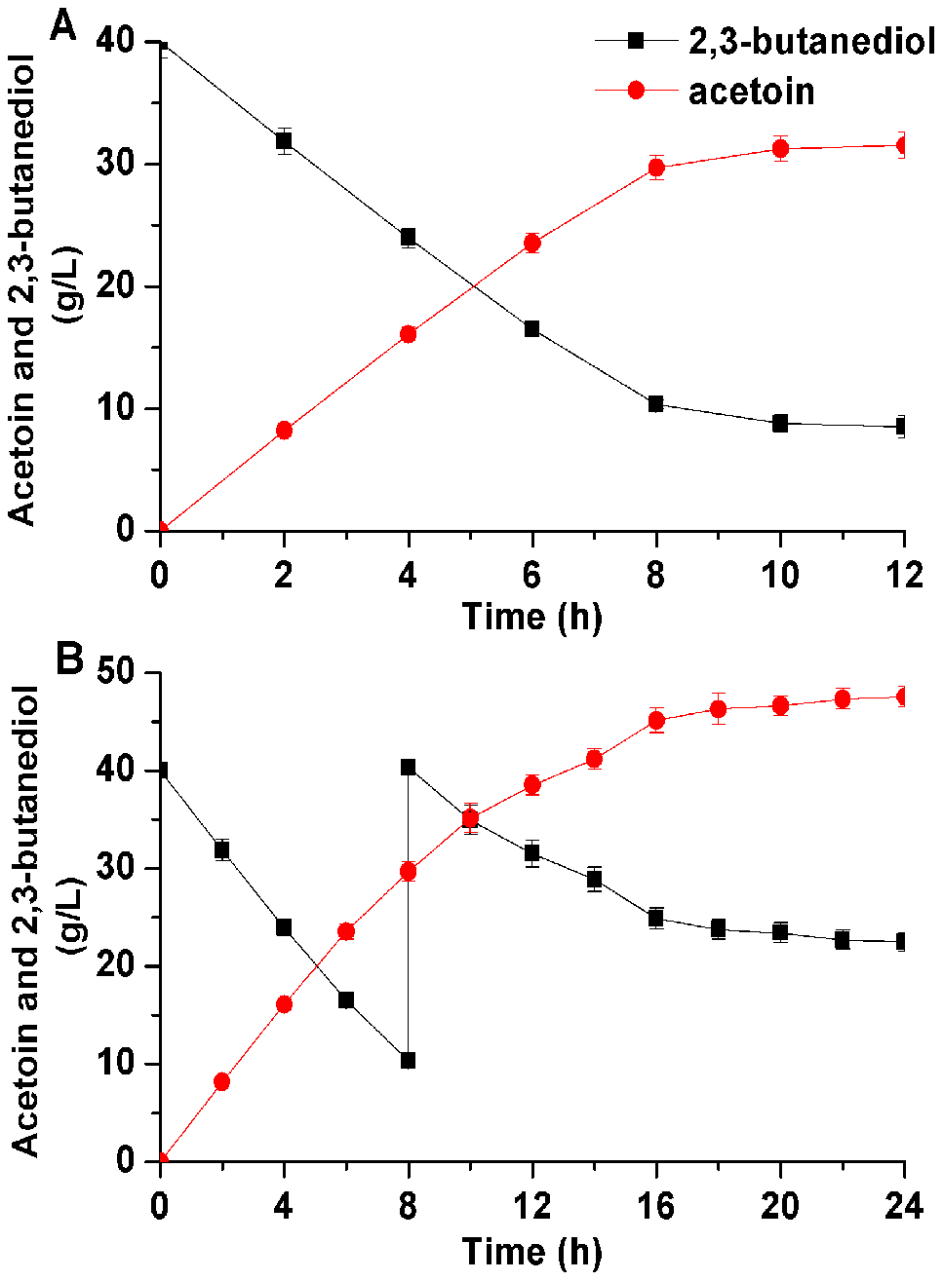
Time course of batch and fed-batch bioconversion of acetoin from 2,3-butanediol. (A) Batch bioconversion; (B) fed-batch bioconversion.

The whole-cell biocatalyst was conducted under optimized conditions as described above in a 5-L fermentor. As shown in Figure 8A, 31.5 g/L AC was acquired from 40.0 g/L 2,3-BD after 12 h with a productivity 2.62 g/(L·h). No other products were detected during the process. Since AR/BDH was a reversible enzyme, the whole-cell biocatalyst could not completely transform 2,3-BD to AC, high concentration of AC would restrain the conversion. As shown in Figure 8B, The initial 2,3-BD concentration was 40.0 g/L, and 30.2 g/L of 2,3-BD was added at 8 h. However, due to the limitation of high concentration of product, the yield of AC could not be effectively increased.

**Figure 9:**
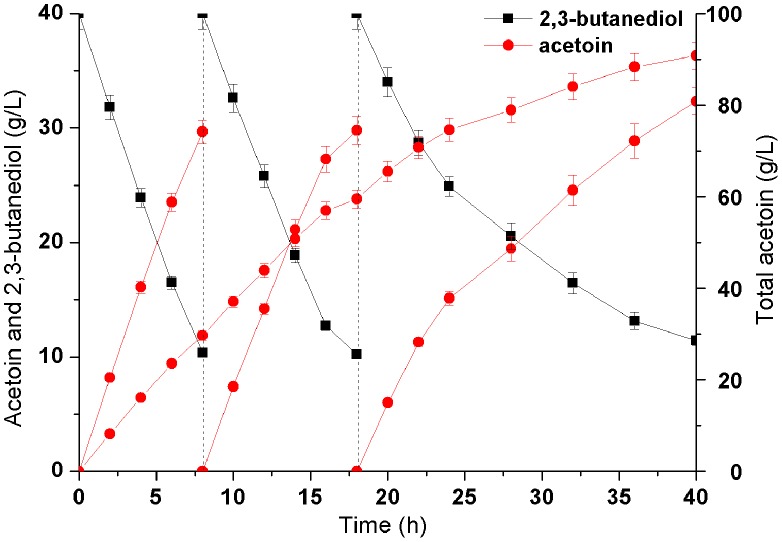
Time course of repeated-batch bioconversion of acetoin from 2,3-butanediol.

To achieve a higher product yield, repeated batch strategies could efficiently enhance the concentrations of the aim products. Due to the NAD^+^ regeneration system, high AC yield could be obtained through more than once whole-cell biocatalyst by using the same batch bacterium without any other additional NADH/NAD^+^. The initial 2,3-BD concentration was 40.0 g/L, after each conversion cycle of 8 h, the cells were harvested and then added by 40.0 g/L of fresh 2,3-BD solution to continue the enzymatic reaction. As shown in Figure 9, there was a stable and efficient increase on AC yield in the first two batch conversion. However, AC productivity gradually decreased in the third batch. Thus, after total 40 h of bioconversion, 91.8 g/L of AC was produced from 120.0 g/L 2,3-BD with the productivity of 2.30 g/(L·h), which was the highest production by biocatalyst (Table 3).

**Table 3:** Comparing of microbial production of acetoin using different fermentative strains or biocatalysts.

Strains	Productivity (g/L · h)	Concentration (g/L)	Yield (mol/mol)	References
**Fermentation**				
* Klebsiella pneumoniae* XZF-308	0.32	25.9	0.16	[42]
* Gluconobacter oxydans* DSM 2003	1.24	89.2	0.91	[10]
* Serratia marcescens* H32	1.88	75.2	0.78	[11]
* Paenibacillus polymyxa* CS107	1.32	55.3	0.76	[8]
* Lactococcus lactis* subsp. *lactis* 3022	0.19	9.28	0.20	[43]
***Bacillus***				
* Bacillus licheniformis* MEL09	1.15	41.3	0.84	[4]
* Bacillus subtilis* CICC10025	0.63	35.4	0.83	[16]
* Bacillus subtilis* 168	0.09	5.5	0.70	[19]
* Bacillus subtilis* 168	0.47	19.8	0.79	[1]
* Bacillus amyloliquefaciens*	1.42	51.2	0.43	[17]
* Bacillus subtilis* JNA-3-10	0.32	42.2	0.57	This lab[15]
* Bacillus subtilis* JNA-3-10-PAR	0.43	41.5	0.71	This lab[18]
* Bacillus subtilis* JNA-UD-6	0.37	53.9	0.74	This lab[14]
**Biocatalyst**				
* Escherichia coli* BL21 (DE3)	3.06	36.7	0.85	[6]
Purified NADPH-dependent carbonyl reductase and glucose dehydrogenase	9.76	12.2	0.85	[5]
* Escherichia coli* Rosetta (DE3)	2.25	13.5	0.91	[44]
* Bacillus subtilis* 168 and *Klebsiella pneumoniae* CICC 10011	1.89	56.7	0.62	[41]
* Bacillus subtilis* 168	2.30	91.8	0.78	This study

This is the first report of applying NAD^+^ regeneration system to produce acetoin in *B. subtilis*. By repeated batch strategy, the whole-cell biocatalyst achieves the purpose of efficient and sustainable producing of AC, and finally reaches the highest AC production record by biocatalysis. In addition, compared to the difficulty of the downstream purification processes of mixed acid-butanediol fermentation, this bioprocess is relatively simple and security, and residual 2,3-BD can be easily separated from AC.

## Conclusions

A biocatalyst for AC production was successfully constructed by introducing the NAD^+^ regeneration system into *B. subtilis*, in which AR/BDH and NOX were co-expressed. After optimization of this converting reaction, repeated batch strategy was further applied on this biocatalyst, and 120.0 g/L 2,3-BD was converted into 91.8 g/L AC with the productivity of 2.30 g/(L·h). To our knowledge, this is the highest report of AC production by biocatalyst. However, AR/BDH stability and the inhibitory effect of acetoin/2,3-butanediol to this enzyme should be further studied, and modeling of this enzyme and site directed mutation is now undergoing. This work proposed an efficient approach for nature AC production and microbial-based biofuel utilization of 2,3-BD.
